# Techniques for Management of Portal Vein Thrombosis during Liver Transplantation

**DOI:** 10.1155/2020/8875196

**Published:** 2020-08-28

**Authors:** Navdeep Singh, Kenneth Washburn, Sylvester Black, Austin Schenk

**Affiliations:** Division of Transplantation, Department of Surgery, The Ohio State University, Wexner Medical Center, Columbus, OH, USA

## Abstract

Portal vein thrombosis (PVT) poses a unique challenge in liver transplant. The management of PVT differs according to the extent of thrombosis. Anastomosis of a donor portal vein to a varix is a viable option when an adequate size varix is identified on preoperative imaging or intraoperatively. Here, we describe our experience in two liver transplant cases with cavernous transformation of the portal vein where the donor portal vein was anastomosed to a varix using a donor iliac vein interposition graft.

## 1. Introduction

In 1985, Shaw and colleagues described successful liver transplantation in seven patients with severely reduced or absent portal flow, and since that time, portal vein thrombosis (PVT) has no longer been considered an absolute contraindication to liver transplantation [1]. PVT is reported in 5-26% of liver transplants [2]. As fibrosis develops in the failing liver, antegrade portal flow is impeded, hepatofugal flow develops, and varices mature. Acute PVT is typically symptomatic, but chronic PVT remains asymptomatic as collateral varices slowly develop over time. Preoperative ultrasound and/or three-dimensional imaging is used to detect PVT and facilitate operative planning. Yerdel and colleagues [3] proposed an anatomic classification system which defined aberrancies in portal flow as (a) <50% occlusion of the portal vein, (b) >50% occlusion of the portal vein (including complete occlusion), (c) complete thrombosis of both the portal vein and proximal superior mesenteric vein (SMV), and (d) complete thrombosis of the portal vein and both the proximal and the distal SMV. Each of these scenarios requires advanced planning and creative surgical techniques. Here, we describe our recent experience with two cases of complete PVT (so-called cavernous transformation) in which alternate portal inflow was established from large intra-abdominal varices (varicoportal anastomosis, VPA).

### 1.1. Case 1

**Figure 1:**
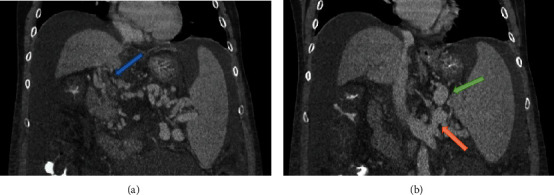
Cross-sectional imaging demonstrating (a) cavernous transformation (blue arrow) and (b) splenorenal shunting (orange arrow) with large upper abdominal varices used for alternate portal inflow (green arrow).

Our first patient was a 58-year-old male with primary biliary cirrhosis and nonalcoholic steatohepatitis. Portal flow was not visualized on preoperative Doppler ultrasound. Contrast enhanced CT demonstrated complete PVT with cavernous transformation (Figure 1(a)) and clot extending into the distal SMV. A large splenorenal shunt and large upper abdominal varices were noted (Figure 1(b)). Intraoperatively, a large left upper quadrant varix was identified as the target portal inflow vessel prior to the hepatectomy. As predicted, the portal vein was completely atretic and without flow, and there were numerous collaterals in the porta hepatis. Once anhepatic, a Satinsky clamp was placed on the target varix and a segment of donor ileac vein was sewn end-to-side to the varix using 6-0 Prolene. No attempt was made to encircle or otherwise dissect the varix. When the anastomosis was complete, the vein graft was occluded distally and the Satinsky was removed. Flow in the conduit was satisfactory, but to further augment portal flow, the left renal vein was ligated. Implantation was then completed with end-to-end anastomosis of the ileac vein jump graft to the donor portal vein. Allograft function was excellent.

### 1.2. Case 2

**Figure 2:**
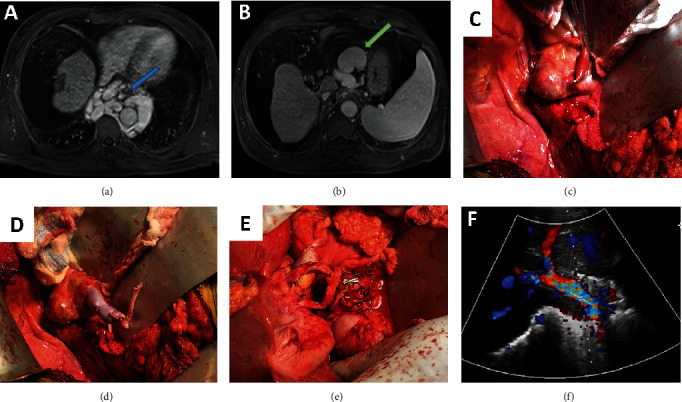
MRI, intraoperative, and ultrasound imaging demonstrating (a) extensive esophageal varices (blue arrow), (b) a large perigastric varix used for portal inflow (green arrow), (c) the clamped varix, (d) an ileac vein graft anastomosed to the varix, (e) the porta postimplantation with varicoportal inflow and the overlying hepatic artery, and (f) Doppler imaging of the varicoportal inflow.

Our second case was a 61-year-old male with cryptogenic cirrhosis and hepatocellular carcinoma. Preoperative imaging revealed cavernous transformation with numerous portal venous collaterals most prominent in the paraesophageal (Figure 2(a)) and perigastric regions (Figure 2(b)). The left renal vein was nondilated, and a splenorenal shunt was not present. Variant portal inflow was created by joining an ileac vein graft to a large perigastric varix (Figures 2(c)–2(f)). Portal flow and hepatic function were excellent.

## 2. Discussion

Portal vein thrombosis can range from mild partial nonocclusive thrombus to complete occlusion with cavernous transformation, and varied surgical techniques are required to handle each scenario [4]. Quality preoperative ultrasound and three-dimensional imaging are essential for operative planning. In cases where PV thrombosis is known preoperatively, we deliberately select younger brain dead donors with minimal steatosis for these recipients, and we utilize the following options to establish portal inflow:

1. Intraoperative portal vein thrombovenectomy
2. SMV jump grafting
3. Establishing renal-portal inflow from left renal vein and donor ileac vein conduit
4. Varix-to-portal anastomoses with or without conduit
5. Portacaval transposition

In all cases, we consider left renal vein ligation or ligation of other collateral vessels to enhance portal inflow. Preoperative IR-guided portal vein recanalization has been described [5], as has arterialization of portal inflow [6], though we have not utilized these techniques in our practice. We have combined all of the stated variants with piggyback implantation technique, and once robust portal inflow is established, it has not been our practice to use postoperative anticoagulation aside from low-dose aspirin.

Physiologic portal inflow is associated with better 1-, 5-, and 10-year outcomes than any alternative described [7], and therefore, PV thrombectomy and primary anastomosis are the procedures of choice whenever possible [8]. When the entire portal vein is unusable, dissection can be carried to the junction of the SMV and splenic veins [9]. When the thrombus extends beyond the splenomesenteric junction, a venous jump graft is sewn to the main trunk of the SMV. When the SMV is unusable, a high flow splenorenal shunt may allow for renal-portal anastomosis. Ideal portal inflow is described as 100 mL of flow per minute per 100 gm of liver tissue, and renal-portal techniques have been reported to provide up to 900 mL/min of flow [10, 11]. When renal-portal anastomosis is not possible, consideration is given to VPA. Use of pericholedochal and peripancreatic varices has been reported [12–14]. Alexopoulos and colleagues report 100% patient survival and good allograft function in 5 patients treated with VPA with a median follow-up of 2.3 years [15]. Portacaval transposition and arterialization of the portal vein are considered last resort measures and are frequently associated with need for retransplantation.

In summary, successful surgical management of PVT requires preoperative imaging, thoughtful planning, surgical creativity, and adherence to a practice-based algorithm for establishing portal inflow.
